# Medical Society of Kentucky

**Published:** 1854-10

**Authors:** 


### MEDICAL SOCIETY OF KENTUCKY.

We hope our readers in Kentucky will not forget, that the State Medical Society is to meet at Covington, on Wednesday, the 25th of October. The last annual meeting was not well attended, and it becomes the more necessary for those who feel an interest in the success and perpetuity of the Society to exert themselves to secure a good attendance at the approaching meeting. No one can doubt the utility of such associations, and those who have been present at former meetings of the Society will testify to the interest that has characterized its proceedings. We owe it to our profession, and to the character of the State, to sustain our Society, which opened under auspices so encouraging. The preparation made by committees authorizes the belief that the meeting in Covington will be one of the most interesting yet held. We hope the attendance will be large.

